# 30ièmes Journées Franco-Belges de Pharmacochimie

**DOI:** 10.3390/ph9040073

**Published:** 2016-11-18

**Authors:** Samia Aci-Sèche, Frédéric Buron, Karen Plé, Laurent Robin, Franck Suzenet, Sylvain Routier

**Affiliations:** Institut de Chimie Organique et Analytique, CNRS UMR 7311, Université d’Orléans, F-45067 Orléans, France; samia.aci@cnrs-orleans.fr (S.A.-S.); frederic.buron@univ-orleans.fr (F.B.); karen.ple@univ-orleans.fr (K.P.); laurent.robin@univ-orleans.fr (L.R.); franck.suzenet@univ-orleans.fr (F.S.)

**Keywords:** organic and medicinal chemistry, chemoinformatics, pharmacochemistry, pharmacology, target discovery, medical imagery

## Abstract

The “Journées Franco-Belges de Pharmacochimie” is a recognized annual meeting in organic and medicinal chemistry known for the quality of scientific exchange and conviviality. Young researchers were encouraged to present their work and share ideas with senior scientists. Abstracts of plenary lectures, oral communications, and posters presented during the meeting are collected in this report.

## 1. Aim and Scope of the Meeting

The “Journées Franco-Belge de Pharmacochimie” is a recognized annual meeting in medicinal chemistry. This 30th edition was held in Amboise located in the “Centre—Val de Loire” Region of France. The goal of this symposium is to promote scientific exchanges between medicinal chemists mainly from France and Belgium. The JFB are renowned for advanced science, conviviality, and outstanding opportunities for senior and young scientists to exchange knowledge. The scientific program included one “General Public” conference, one opening conference and seven plenary lectures by internationally recognized scientists. Industrial and academic scientists gave presentations mainly in imaging (TEP), oncology and CNS disorders. An important part of the program was devoted to open lectures (13 oral communications) giving the younger scientists an opportunity to present their research.

## 2. Conferences

### 2.1. Wine and Chemistry

BouyssouPascalInstitut de Chimie Organique et Analytique, UMR CNRS 7311, Université d’Orléans, F-45067 Orléans Cedex 2, France; pascal.bouyssou@univ-orleans.fr

Chemistry and wine, two words that seem diametrically opposed in the public opinion, and yet wine is chemistry! Together during this “aperitif conference,” we’ll walk through vineyards and cellars to discover a small part of the chemistry from grapes to wine.

### 2.2. Marine Imidazolinone Alkaloids as a Source of Inspiration for the Synthetic Development of Kinase Inhibitors: A Case Study in Heterocyclic Study

BazureauJean-Pierre^1^*BurgyGuillaume^1^,^2^LimantonEmmanuelle^1^GuihéneufSolène^1^CarreauxFrançois^1^TahtouhTania^2^,^3^DurieuEmilie^2^,^3^MeijerLaurent^2^1Institut des Sciences Chimiques de Rennes ISCR UMR CNRS 6226, Université de Rennes 1, groupe ICMV, Bât. 10A, Campus de Beaulieu, CS 74205, 263 Av. du Gén. Leclerc, F-35042 Rennes Cedex, France2ManRos Therapeutics, Perharidy Research Center, Centre Médical de Perharidy, F-29680 Roscoff, France3Station Biologique CNRS, *KISSf* platform, Place George Teissier, F-29680 Roscoff, France*Correspondence: jean-pierre.bazureau@univ-rennes1.fr

The synthetic development of stereo-controlled (5*Z*)-5-arylideneimidazolinones as medicinal platforms began in our laboratory in the 1990s. In the first period, the story begins with the construction of these platforms by 1,3-dipolar cycloaddition reactions based on aminoester imidate ylides (Lerestif, J.-M., et al. *Tetrahedron* 1995, *51*, 6757–6774). The presence of an amino group in position C-2 of the imidazolinone platform showed the limits of these 1,3-dipolar cycloadditions. This fact led us, in a second period, to thoroughly explore the thiohydantoin approach (Cherouvrier, J.-R., et al. *Green Chem.* 2001, *3*, 165–169) to build various marine alkaloid derivatives, i.e., leucettamine B (Cherouvrier, J.-R., et al. *Tetrahedron Lett.* 2002, *43*, 3581–3584), dispacamide A (Guihéneuf, S., et al. *Org. Biomol. Chem.* 2012, *10*, 978–987) as (5*Z*)-2-amino-5-arylideneimidazolin-4-ones or analogs of dispacamide A bearing rhodanine platforms (Guihéneuf, S., et al. *Mol. Divers.* 2014, *18*, 375–381; *Curr. Microwave Chem.* 2014, *1*, 33–40) by using three-component reaction (Renault, S., et al. *J. Comb. Chem.* 2007, *9*, 935–942) and sulfur/nitrogen displacements under microwave irradiation (Debdab, M., et al. *Heterocycles* 2009, *78*, 1191–1203).


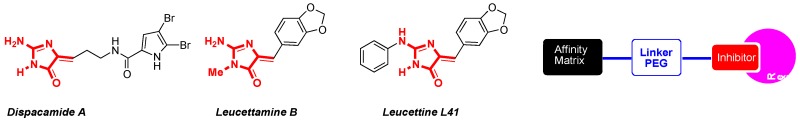


Then, in the third period, the controls of these synthetic methods highlighted that leucettamine B analogs, named leucettines (Debdab, M., et al. *J. Med. Chem.* 2011, *54*, 4172–4186; Renault, S., et al. Patent WO 2009/05032 (A2) 23 April 2009), present an excellent capacity of inhibition of the protein kinase DYRK1A. In the leucettine library, leucettine L41 appeared as a good inhibitor of this protein kinase (DYRK1A IC_50_ 15 nM), which is directly connected to Alzheimer’s disease (AD) and Down’ syndrome (Fant, X., et al. *Mol. Pharmacol.* 2014, *85*, 441–450; Nert, G., et al. *Eur. Neuropsychopharm.* 2015, *25*, 2170–2182). Starting from cocrystallized structure of L41 with DYRK1A, we shall show that immobilization of one of the leucettines (leucettine L41) by affinity chromatography (Burgy, G., et al. *Eur. J. Med. Chem.* 2013, *62*, 728–737) on agarose gel is a relevant approach from cell extracts of mouse brain (Tahtouh, T., et al. *J. Med. Chem.* 2012, *55*, 9312–9330).

Finally, the story will end, in the fourth period, with the presentation of the roadmap for the preparation of the next pre- and clinical trials in order to validate a potential drug for AD.

**Acknowledgments:** This research was supported by grants from the ‘Fonds Unique Interministériel” (FUI) PHARMASEA and TRIAD projects, the “Association France-Alzheimer (Finistère)” (LM) and “Fondation Jérôme Lejeune” (LM). JPB’s funding for the leucettine project included “CRITT Santé Bretagne” and “CBB Développement Bretagne.” We are thankful to C. BENESTEAU and L. BERNARD-TOUAMI (Animalerie de l’Université de Rennes 1) for providing the mouse tissues. Guillaume BURGY is recipient of a “CIFRE” PhD fellowship. Solène Guihéneuf and Tania TAHTOUH are recipients of a PhD fellowship from the “Ministère de la Recherche et de la Technologie” (MRT).

### 2.3. Galactosylceramides and Cancer Immunotherapy. Synthesis, Vectorization and Biological Evaluation

PipelierMurielCEISAM, Chimie et Interdisciplinarité, Synthèse, Analyse, Modélisation—UMR CNRS 6230-2, rue de la Houssinière, BP 92208, F-44322 NANTES Cedex 3, France; muriel.pipelier@univ-nantes.fr

CD1d-restricted T lymphocytes, a subclass of human lymphocytes, appear to be major players of the immune response. A subpopulation of lymphocytes, iNKT cells, bears a TCR receptor and reacts against α-galactosylceramides (α-GalCer) (Natori, T., et al. *Tetrahedron Lett.* 1993, *34*, 5591–5592). Recognition of α-GalCer bound to CD1d molecule of an antigen-presenting cell, leads to the fast and strong secretion of a large panel of cytokines. These cytokines can stimulate the maturation of dendritic cells, activate the proliferation of IFNγ synthesis and stimulate cytotoxic CD8 lymphocytes. These mechanisms contribute to the control of tumor progression (TH1). iNKT cells can also act as suppressors of the immune response through their secretion of cytokines such as IL-4 (TH2). The α-anomeric configuration is required for the full activity and this point is quite surprising as galactosylceramides have β configuration in mammals. SAR studies and chemical modifications on the ceramide moiety led to the discovery of the first analog under clinical trials as anticancerous drug, namely KRN7000 (Wilson, M.T., et al. *Trends Mol. Med.* 2002, *8*, 225–231; Van Kaer, L., *Nat. Rev. Immunol.* 2005, *5*, 31–42). However, the KRN7000 presents some major drawbacks: low availability; a concomitant activation of TH1/TH2 leading to side effects, especially an anergy of the iNKT; poor solubility; and low bioavalability (Parekh, V.V., et al. *J. Clin. Investig.* 2005, *115*, 2572–2583).


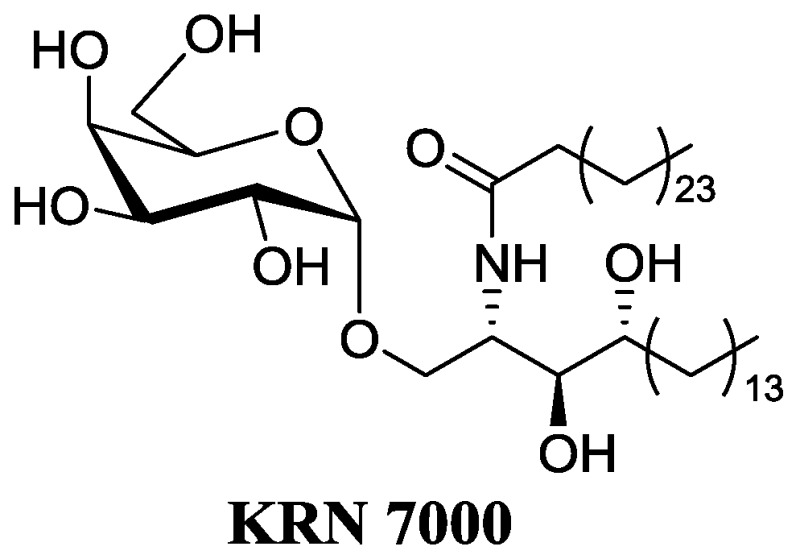


Therefore, we focus on the synthesis of deoxy α-GalCer analogs which would be as potent as KRN7000 but with less side effects and drawbacks. The effect of these modifications on the TH1/TH2 balance has been evaluated in vitro on human cells (ISERM) and in vivo on mice, (CHU Nantes—plate forme de transfert du Cancéropole Grand Ouest) (Dubreuil, D., et al. World Patent WO2008047249 (A2); Lacône, V., et al. *J. Med. Chem.* 2009, 52, 4960–4963; Hunault, J., et al. *J. Med. Chem.* 2012, *55*, 1227–1241). Some analogs have shown promising results for cancer immunotherapy.

**Acknowledgments:** This work was supported by the ANR, “la fondation ARC pour la recherche sur le cancer” and “la ligue contre le cancer.”

### 2.4. In Vivo Preclinical Molecular Imaging: From the Laboratory Bench to the Patient

PlenevauxAlainGIGA-CRC In vivo Imaging, University of Liege, Allée du six août 8, B-4000 Liège, Belgium; alain.plenevaux@ulg.ac.be

Preclinical molecular imaging plays a key role in the study of diseases and the development, evaluation, andvalidation of novel treatment and diagnostic techniques. The actual aim is to facilitate the translation of innovative therapies and diagnostic agents into patients by providing in vivo proof-of-principle data in animal models of diseases and accordingly match some of the legal requirements before human clinical trials. The real power of preclinical imaging stems from the fact that in vivo data are non-invasively collected, thus making longitudinal studies possible. The evolution of the disease or the actual impact of innovative treatments can be evaluated in the same animal over a long period of time. The major modalities used in preclinical molecular imaging (all present in our laboratory) are the positron emission tomography (microPET), the X-ray tomodensitometry (microCT) and the magnetic resonance imaging (microMRI). The GIGA-CRC In vivo Imaging at ULg is a multidisciplinary laboratory which relies on a medical cyclotron for radioisotope productions, a radiochemistry unit for the development of new radiopharmaceuticals and process automation, a GMP unit for radiopharmaceutical productions, a preclinical imaging unit and a clinical imaging platform (with MRI, PET, EEG, TMS, …). All expertise and qualified staff are present on site. Our laboratory has all necessary permission and approval for radiopharmaceutical productions and human clinical trials. During this presentation, we will take as an example the development of the first SV2A radioligand [^18^F]UCB-H (a-b) to illustrate how we can bring new ideas from the laboratory bench to the patient (Bretin, F., et al. *Eur. J. Nucl. Med. Mol. Imaging Res.* 2013, *3*, 35; Warnock, G., et al. *J. Nucl. Med.* 2014, *55*, 1336–1341).


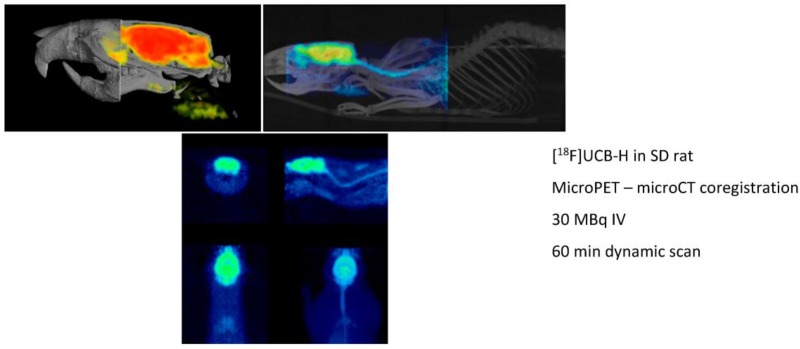


## 3. Oral Communications

### 3.1. Synthesis of Polyfunctionalized Imidazo[2,1-b][1,3,4]thiadiazoles as Novel DYRK1A/CLK1 Dual Inhibitors

PlaceMatthieuCopinChloéBuronFrédéricRoutierSylvain*Institut de Chimie Organique et Analytique, CNRS, UMR 7311, Université d’Orléans, B.P. 6759, F-45067 Orléans Cedex 2, France*Correspondence: sylvain.routier@univ-orleans.fr

The interest in the imidazo[2,1-*b*][1,3,4]thiadiazole (Terzioglu, N., et al. *Eur. J. Med. Chem.* 2003, *38*, 781–786; Hoelzmann, G., et al. WO2010/12345A1; Khazi, I.A.M., et al. *Tetrahedron*, 2011, *67*, 3289–3316) moiety for application in pharmaceutical products makes this scaffold a highly useful building block for organic chemistry. Such derivatives have found applications in oncology (Romagnoli, R., et al. *Eur. J. Med. Chem*. 2015, *101*, 205–217), infectiology (Alwan, W.S., et al. *Eur. J. Med. Chem*. 2015, *95*, 514–525) or neurodegenerative diseases (Patel, H.M., et al. *Eur. J. Med. Chem*. 2015, *93*, 599–613).

However, the synthetic tools for accessing highly functionalized imidazothiadiazoles are very limited, and only few functionalization methods are described (Chiang, H.A., et al. *Org. Lett*. 2007, *9*, 1449–1451; Capriati, V., et al. *Eur J. Org. Chem.* 2002, *3*, 478–484; Kim, S.H., et al. *Org. Lett*. 2010, *12*, 1868–1871). In order to increase the molecular diversity of these derivatives, there is consequently tremendous interest in developing efficient synthetic methodologies.

Consequently, we developed several methodologies to modulate regioselectively the C-2, C-5 and C-6 positions of this scaffold (Copin, C., et al. *Eur. J. Org. Chem*. 2012, 3079–3083; Copin, C., et al. *Eur. J. Org. Chem.* 2015, 6932–694; Copin, C., et al. *Synlett* 2016, *27*, 1091–1095; Copin, C., et al. *Eur. J. Org. Chem.* 2016, 1958–1962). In order to create C–C, C–N, C–O or C–S bonds, we used various reactions such as S*_N_*Ar, C–H arylation, and palladium-catalyzed cross-coupling. We investigated the reactivity of each position and showed the influence of previously introduced groups.

Finally, we implemented these efficient methodologies to design dual inhibitors. Indeed, the functionalized scaffold could be a strong inhibitor of DYRK1A and CLK1 kinases, involved in the neuronal degeneration pathway observed especially in Alzheimer’s disease.

These methodologies, the synthesis of the compounds and the results of biological tests will be presented in this communication.


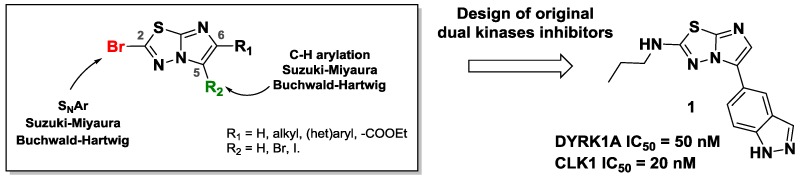


### 3.2. Synthesis of Pentamidine Analogs Bearing Heterocyclic Platforms and Their Biological Evaluations

AmbeuChristelle^1^,^2^*BazureauJean-Pierre^1^CoulibalyWacothon Karime^1^,^2^PaquinLudovic^1^Mamyrbékova-BékroJanat^2^BékroYves-Alain^2^BéniéAnoubilé^2^Le GuévelRémy^3^CorluAnne^3^BachStéphane^4^RuchaudSandrine^4^1Institut des Sciences Chimiques de Rennes ISCR UMR CNRS 6226, Université de Rennes 1, groupe ICMV, Bât. 10A, Campus de Beaulieu, CS 74205, 263 Av. du Gén. Leclerc, F-35042 Rennes Cedex, France2Laboratoire de Chimie Bio Organique et des Substances Naturelles (LCBOSN), Université Nangui Abrogoua, BP 802, Abidjan 02, République de la Côte d’Ivoire3ImPACcell Platform, SFR Biosit, Université de Rennes 1, Bât. 8, 2 Avenue du Prof. Léon Bernard, CS 34317, F-35043 Rennes Cedex, France4Station Biologique CNRS, *KISSf* platform, Place George Teissier, F-29680 Roscoff, France*Correspondence: christelle.ambeu@univ-rennes1.fr

Our work is focused on the development of multi-step synthesis strategy of new compounds bearing several heterocyclic platforms (rhodanine, benzimidazole (Brugidou, R., et al. *Heteroatom Chem.* 1999, *10*, 447–454), pyrazole and imidazole) for multiple therapeutic uses to fight malaria, leishmaniasis, cancer and neurodegenerative diseases. Pharmacomodulations of these compounds were developed from the design of pentamidine, which contains two benzamidine moieties (“Western” and “Eastern” sides).


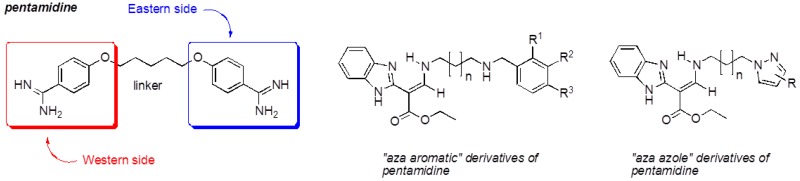


Indeed, the substitution of its “Western” side by a platform rhodanine or benzimidazole and its “Eastern” side by an “azole” system (pyrazole, imidazole) led respectively to 5-arylidenerhodanines (Ambeu, C., et al. *Curr. Microw. Chem.* 2016, *3*, 145–156; *Med. Chem. Res.* 2016, submitted), to “aza” derivatives and to “aza-azole” derivatives, which are pentamidine analogs. These compounds were evaluated for their antiproliferative activity on tumoral cell lines and for their inhibitory activity on protein kinases.

**Acknowledgments:** One of us (C.A.) wishes to thank the “ElectBTP” and the “Ministère de l’Enseignement Supérieur et de la Recherche de la Côte d’Ivoire” for the grant. Financial support of this program carried out under the French National Cancer Institute “Cancéropôle Grand Ouest” by contract “Ion Channel-Network CGO 2012,” is gratefully acknowledged.

### 3.3. Synthesis of Analogs of SKF-96365 for the Modulation of SOCEs

DagoCamille Déliko^1^,^2^*PaquinLudovic^1^Mamyrbekova-BékroJanat Akhanovna^2^BékroYves-Alain^2^RuchaudSandrine^3^Le GuévelRémy^4^BrigaudeauChristophe^5^MignenOlivier^6^BazureauJean-Pierre^1^1Institut des Sciences Chimiques de Rennes ISCR UMR CNRS 6226, groupe ICMV, Université de Rennes 1, Bât. 10A, Campus de Beaulieu, CS 74205, 263 Av. du Gén. Leclerc, F-35042 Rennes Cedex, France2Laboratoire de Chimie Bio Organique et des Substances Naturelles (LCBOSN), Université Nangui Abrogoua, BP 802, Abidjan 02, République de la Côte d’Ivoire3Station Biologique CNRS, *KISSf* platform, Place George Teissier, 29680 Roscoff, France4ImPACcell Platform, SFR Biosit, Université de Rennes 1, Bât. 8, 2 Avenue du Prof. Léon Bernard, CS 34317, F-35043 Rennes Cedex, France5CalciScreen Platform, Université de Bretagne Occidentale, 22 avenue Camille Desmoulins-CS 93837, F-29238 Brest Cedex 3, France6Laboratoire Canalopathies et Signalisation Calcique, Inserm U1078, Université de Bretagne Occidentale, 22 Avenue Camille Desmoulins, F-29200 Brest Cedex, France*Correspondence: deliko.dago@univ-rennes1.fr

Disturbances in calcium signaling (Ca^2+^) in the development of leukemia have been studied extensively, and reveal their importance in various stages of disease progression ranging from the control of cell differentiation; to the resistance to apoptosis and therapeutic response; to clinical evolution. The chronic lymphoid (CLL) and the myeloid leukemia (CML) are characterized by Ca^2+^ entry deregulations and especially an altered store-operated Ca^2+^ entry (SOCE) in which the Orai Ca^2+^ channel is very important (Lodola, F., et al., *PLoS ONE* 2012, *7*, e42541). A defection of SOCE has also been associated with other types of cancers such as prostate and breast cancer (Flourakis, M., et al., *Cell Death Dis.* 2010, *1*, e75; Motiani, R.K., et al., *J. Biol. Chem.* 2010, *285*, 19173–19183).

It is important to note that there is currently no specific modulator of ion channels mediating Ca^2+^ entries in non-excitable cells. The scientific community, however, considers the discovery of Ca^2+^ entry modulators, easily accessible to chemical modulation, as a potentially important advance in cancer therapy that could lead to a new class of anti-tumor agents.

In this context, we are interested in SKF-96365 (Dago, C.D., et al. *Molbank* 2016, *2016*, submitted). Originally identified as a blocker of receptor-mediated calcium entry, it is widely used diagnostically as a blocker of transient receptor potential canonical type (TRPC) channels (Merrit, J.E., et al., *J. Pharmacol.* 1989, *98*, 674P; Merrit, J.E., et al. *Biochem. J.* 1990, *271*, 515–522). We have developed analogs of SKF-96365 as potential modulators of the Orai Ca^2+^ channel, in order to establish a relationship activity (RSA) study. Results from preliminary work will be presented.


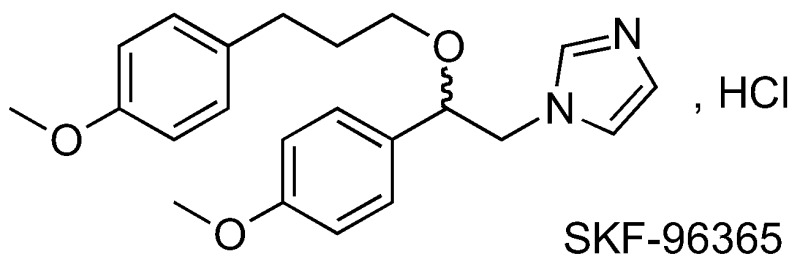


**Acknowledgments:** One of us (C.D.D.) wishes to thank the “Benian International Fondation” and the “Ministère de l’Enseignement Supérieur et de la Recherche de la Côte d’Ivoire for the grant. Financial support of this program carried out under the French National Cancer Institute “Cancéropôle Grand Ouest” by contract “Ion Channel-Network CGO 2012,” is gratefully acknowledged.

### 3.4. Frags2Drugs: A Novel in Silico Tool for Fragment-Based Lead Discovery

GallyJosé-ManuelHeinrichJean-ThomasObledAlanAci-SècheSamiaBonnetPascal*Bioinformatique Structurale et Chémoinformatique, Institut de Chimie Organique et Analytique (ICOA), UMR CNRS-Université d’Orléans 7311, Université d’Orléans BP 6759, F-45067 Orléans Cedex 2, France*Correspondence: pascal.bonnet@univ-orleans.fr

During the past decade, fragment-based lead discovery (FBLD) and drug design (FBDD) approaches have been successful in identifying promising compounds for drug research and development. The first kinase-targeted drug approved by the FDA and originated from FBDD is vemurafenib (Bollag, G., et al. *Nat. Rev. Drug Discov.* 2012, *11*, 873–886.), targeting unresectable or metastatic melanoma for patients bearing BRAF V600E mutation. Since then, FBDD has been extensively studied and is currently applied in many drug discovery projects.

Fragments—small molecules with low molecular weight (<300 Da) and flexibility—bind weakly to the receptor but form more efficient interactions in comparison to hits identified by high throughput screening HTS. By linking multiple specific fragments together within the active site of a protein kinase, designing potent and selective inhibitors becomes promising.

We present here a new in silico FBLD tool, Frags2Drugs, that combines both structural information of the fragments within the binding site of protein kinases and their corresponding interaction scores computed by a docking tool. Positions of the fragments within the active site can be extracted from co-crystalized ligands or obtained by molecular docking. The fragments selected by a user-defined location within the active site are then flagged as starting points (seeds) for the fragment-linking step while the atoms of the remaining fragments, suitable for making new bonds, are tagged as growing atoms. The fragments forming steric clashes with the receptor are discarded. Additionally, information of structural solvent molecules can be included during the fragment-growing process.

Once these preliminary steps have been completed, the fragment-linking task is performed to connect seeds and non-seed fragments on specific growing atoms using predefined criteria. Several in silico scores (“Score Efficiency”) are computed to identify and prioritize the most promising designed molecules. We validated this tool by identifying known kinase inhibitors.


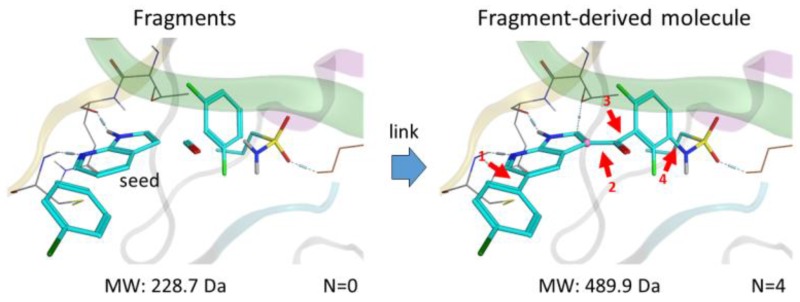


### 3.5. Design and Synthesis of YAP-TEAD Complex Inhibitors as New Anticancer Drugs

GibaultFloriane*CorvaisierMatthieuBaillyFabriceHuetGuillemetteMelnykPatriciaCotellePhilippeOnco et NeuroChimie, Centre de Recherche Jean Pierre Aubert (JPArc), UMR-S 1172, INSERM, Université de Lille, Centre Hospitalier Régional Universitaire (CHRU), F-59000 Lille, France*Correspondence: floriane.gibault@ed.univ-lille1.fr

Recently, the Hippo pathway has been demonstrated to play a crucial role in the organ size control by regulating the phosphorylation of the yes-associated protein 1 (YAP), a transcriptional coactivator with oncogenic activity (Johnson, R., et al. *Nat. Rev. Drug Discov.* 2014, *13*, 63–79). When YAP is phosphorylated, it is sequestrated in the cytoplasm or degraded. When YAP is not phosphorylated, it can enter into the nucleus and interacts with TEAD, a transcription factor, to form YAP-TEAD complex that activates the gene expression in charge of cell proliferation and apoptosis. Overexpression of YAP in several cancers disrupts the balance and urges on YAP-TEAD complex formation causing excessive proliferation and metastasis. Inhibiting this complex formation is thus a promising therapeutic target for the design of new anti-cancer drugs (Li, Z., et al. *Genes Dev.* 2010, *24*, 235–240).

To inhibit the interaction between YAP and TEAD, two strategies are considered. The first one consists in targeting the YAP protein. Currently, Verteporfin (a) and other porphyrins have been identified as YAP-TEAD complex inhibitors (Liu-Chittenden, Y., et al. *Genes Dev.* 2012, *26*, 1300–1305). The mechanism of action of Verteporfin being still unknown, we have decided to synthesize a series of dipyrrins (b), representing fragments of porphyrins to define the minimal requirement yielding the expected biological activity. A second approach to inhibit the YAP-TEAD interaction consists in targeting the TEAD protein by synthesizing ligands able to fit in specific interface 3 (Pobbati, A.V., et al. *Cancer Biol. Ther.* 2013, *14*, 390–398). Based on the X-raystructure of YAP-TEAD complex (c), virtual screening of commercial libraries allowed us to identify four chemical families. The synthesis of one of them is currently under optimization to establish structure-activity relationships. Biological and binding tests are still in progress but first results have already allowed us to identify a promising compound.


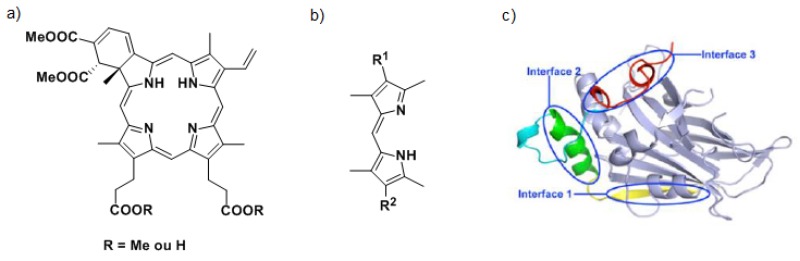


### 3.6. From Design to Pharmacological Evaluation of Benzoxazole Derivatives as Adenosine A_2A_ Receptor Antagonists for the Potential Treatment of Alzheimer’s Disease

DurouxRomain^1^*RenaultNicolas^1^AgouridasLaurence^1^LopesLuisa^2^MelnykPatricia^1^YousSaïd^1^1Onco et NeuroChimie, Centre de Recherche Jean Pierre Aubert (JPArc), UMR-S 1172, INSERM, Université de Lille, Centre Hospitalier Régional Universitaire (CHRU), F-59000 Lille, France2Instituto de Medicina Molecular, 1640-028 Lisbon, Portugal*Correspondence: romain.duroux@univ-lille2.fr

Alzheimer’s disease (AD) is the most prevalent form of dementia in the aged population characterized mainly by the presence of senile plaques and neurofibrillary tangles. Thus far, there is no way to halt or slow down AD. There is thus a constant need for developing novel therapeutic strategies.

The adenosine A_2A_ receptor (A_2A_R), expressed in the CNS, belongs to the class of G protein-coupled receptors (GPCRs). In recent years, A_2A_R has attracted growing interest for AD, where it has been proved that the A_2A_ receptor is over expressed. It has also been found that the A_2A_ receptor antagonists such as caffeine improves memory performance. Though several A_2A_R antagonists have reached clinical trials, most of them suffer from poor pharmacokinetic and pharmacodynamic properties. Current efforts therefore focus on developing new antagonists with relevant Absorption, Distribution, Metabolism, and Excretion ADME properties.

Based on the recently published crystalline structure of the A_2A_R complexed with the selective and high-affinity antagonist triazine and on a pharmacophoric model (Xu, Z., et al. *J. Mol. Model.* 2010, *16*, 1867–1876), we designed new ligands using in silico docking studies (Ia). Our objective is to develop selective A_2A_R antagonists devoid of chemical stability, bioavailability and toxicity drawbacks of compounds currently under clinical trials. Pharmacomodulations of a new benzoxazoles family have been developed (Ib) and show micromolar to nanomolar affinity on HEK293 cells membranes expressing A_2A_R. Cytotoxicity has also been evaluated on SY5Y cells.


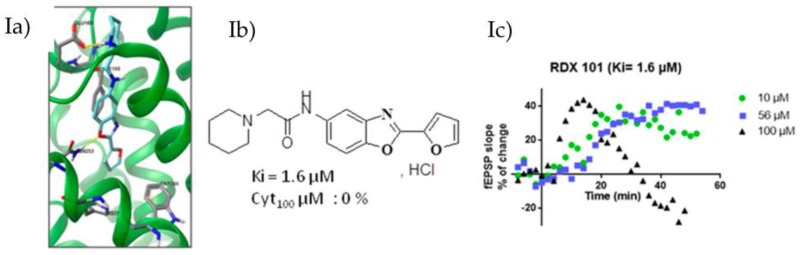


From selected compounds, we evaluated the impact on synaptic transmission by performing hippocampal field excitatory post-synaptic potential recordings (fEPSP) in transgenic rats with a neuronal-specific human A_2A_R overexpression [tg(CAMKII-hA_2A_R)] (Ic).

### 3.7. Tyro3 as a New Therapeutic Target in Bladder Cancer: Molecular Modeling, Synthesis and In Vitro Biological Evaluation

PiguelSandrineUMR9187-U1196/ Institut Curie, Université Paris Sud, Université Paris-Saclay, F-91405 Orsay Cedex, France; sandrine.piguel@curie.fr

Recently, the transmembrane receptor tyrosine kinase Tyro3 has been identified as a new and promising therapeutic target in bladder cancer. (Bernard-Pierrot, F., et al. Patent WO2010/031828-25/03/2010). However, as Tyro3 belongs to the TAM family (Tyro3, Axl and Mer) with a high level of sequence similarity, the design and synthesis of selective inhibitors of Tyro3 remain a challenge to address. Only a few molecules have been specifically designed to inhibit Tyro3 so far, and most of the known inhibitors were actually developed to target other protein kinase targets and then identified through selectivity profiles (Baladi, T., et al. *Eur. J. Med. Chem.* 2015, *105*, 220–237).

As part of a multidisciplinary program, we have designed and synthesized type II potential kinase inhibitors of Tyro3 based on various heterocyclic scaffolds since these type II inhibitors are usually expected to display better selectivity. A library of purine analogs 1 with structural diversity has led to the identification of a potent inhibitor of the TAM family with Kd values of 200, 39 and 42 nM against Tyro3, Axl and Mer respectively. Docking studies of this inhibitor into the modelled DFG-Out structure of Tyro3 revealed a type II binding mode (Suárez, R.M., et al. *Eur. J. Med. Chem.* 2013, *61*, 2–25). Another library of pyrimidine analogs 2 has been synthesized and, analogously, inhibition against Axl was higher than for Tyro3 and Mer (Traoré, T., et al. *Eur. J. Med. Chem.* 2013, *70*, 789–801).


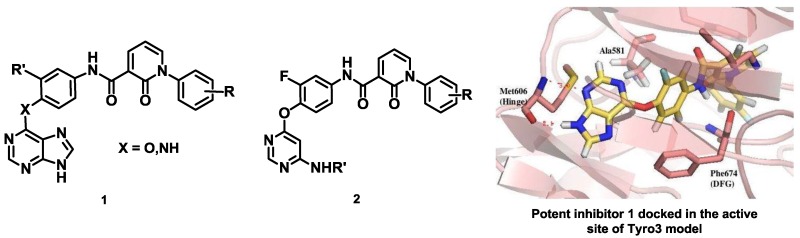


### 3.8. Synthesis of Phenothiazine Derivatives: Alternatives to Manage Antibiotic-Resistant Gram-Negative Bacteria Infections

StutzmannAurélien^1^SchnetterleMarine^2^CaclardArnaud^2^BiotFabrice^2^ValadeEric^2^BollaJean-Michel^1^PagèsJean-Marie^1^BoyerGérard^1^*AlibertSandrine^1^Neulat-RipollFabienne^2^1IRBA, UMR-MD1, Transporteurs Membranaires-Chimiorésistance et Drug-Design, Faculté de Pharmacie et Faculté de Médecine, Aix-Marseille Université, 27 boulevard Jean Moulin, F-13385 Marseille Cedex 05, France2Institut de Recherche Biomédicale des Armées, Unité de Bactériologie/UMR-MD1, DGA MNRBC/PLATLAB, 5 rue Lavoisier, F-91710 Vert le Petit, France*Correspondence: gerard.boyer@univ-amu.fr

Antimicrobial drugs have been crucial tools of health for decades due to their effectiveness in control of bacterial infections. However, soon after their discovery, some pathogens rapidly developed multi-drug resistance (MDR) to antibiotics (Wong, K., et al. *Trends Biochem. Sci.* 2014, *39*, 8–16). The overexpression of efflux mechanisms contributes widely to the MDR phenotype of Gram-negative bacteria. During recent years, investigation of inhibition of MDR attracted worldwide attention (Bolla, J.-M., et al. *FEBS Lett.* 2011, *585*, 1682–1690).

By a medicinal chemistry approach, phenothiazine derivatives were synthesized in order to determine their chemical features as potential adjuvants of usual antibiotics on *Burkholderia pseudomallei*, highly pathogenic Gram-negative bacteria responsible for melioidosis, an endemic disease in Southeast Asia and Northern Australia with a mortality rate of up to 40%, and classified as bioterrorism agents.


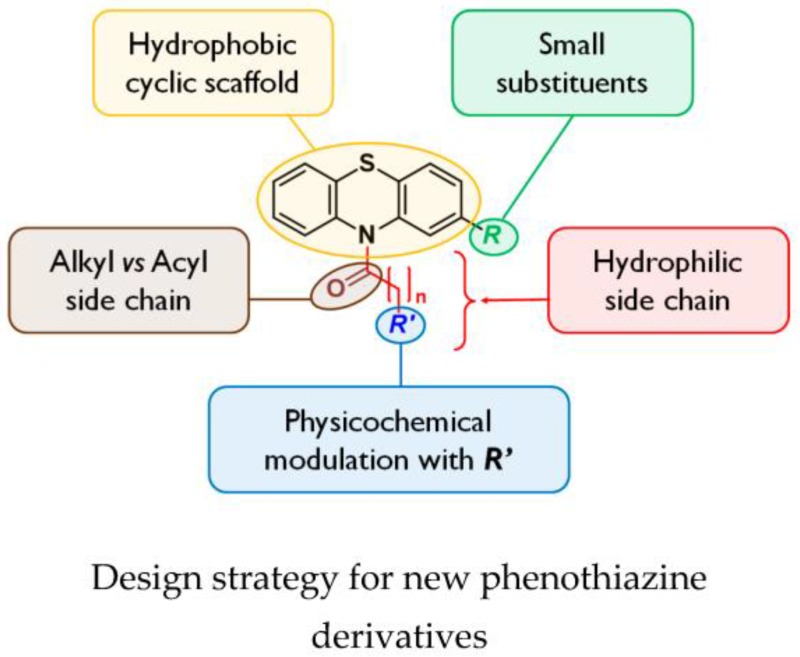


In a first step, the activity of *N*-substituted phenothiazines was determined with various functional groups, using the Epsilometer test method on the non-pathogenic species *Burkholderia thailandensis* (Biot, F., et al. *PLoS ONE* 2013, *8*, e84068); they appear to have no direct activity on bacteria but can potentiate the activity of diverse classes of antibiotics or have an adverse effect on efflux pump expression.

This subject also opens new perspectives for public and militaryhealthcare in the field of the natural or provoked biological risk.

**Acknowledgments:** We thank the Direction Générale de l’Armement (DGA) for the financial support to this project.

## 4. Posters

### 4.1. Conception and Evaluation of Fluorinated Heterocyclic Compounds as COX-2 Ligands to Visualize and Quantify Neuroinflammation by PET

ElieJonathan^1^BuronFrédéric^1^BidaultRudy^2^ArlicotNicolas^2^GuilloteauDenis^2^RoutierSylvain^1^VercouilleJohnny^2^1Institut de Chimie Organique et Analytique, UMR CNRS 7311, Université d’Orléans, Rue de Chartres, BP 6759, F-45067 Orléans, France2CERRP, UMR INSERM U930, Université de Tours, 3 Rue Germaine Richier, F-37100 Tours, France*Correspondence: jonathan.elie@univ-orleans.fr

Central nervous system (CNS) disorders such as multiple sclerosis, stroke, neurodegenerative diseases (Alzheimer’s and Parkinson’s) lead to neuroinflammation that controls the pathology spread and repairs or regenerates the affected tissues. Microglia, the main defense of the CNS, is activated during a neurodegenerative event and leads to production of neuroprotective but also pro-inflammatory factors. This duality of action thereby maintains a vicious circle leading to neuron death. In neuroinflammation processes, cyclooxygenase 2 (COX-2), an enzyme that allows the formation of prostaglandins from arachidonic acid, is strongly overexpressed. Thus, COX-2 appears as a relevant target to early diagnose neuroinflammation with the development of suitable imaging tools (PET tracers).

There are many COX-2 inhibitors currently on the market and those we have chosen as a model are part of the class of NSAIDs and particularly coxibs to get a specific ^18^F COX-2 tracer. Nowadays, no compound has a sufficient biodistribution in the CNS, limiting their potential as future radiotracers targeting COX-2.

We therefore proposed to synthesize new specific inhibitors of COX-2, based on original skeleton type of (aza)indazoles substituted with groups that we believe will be in the heart of interactions with their target. The biological evaluations were performed using the COX Fluorescent Inhibitor Screening Assay Kit (Cayman, item No. 700100). The second part of our work was to develop minutes reaction to introduce ^18^F (half-life ^18^F = 110min) on lead compounds.


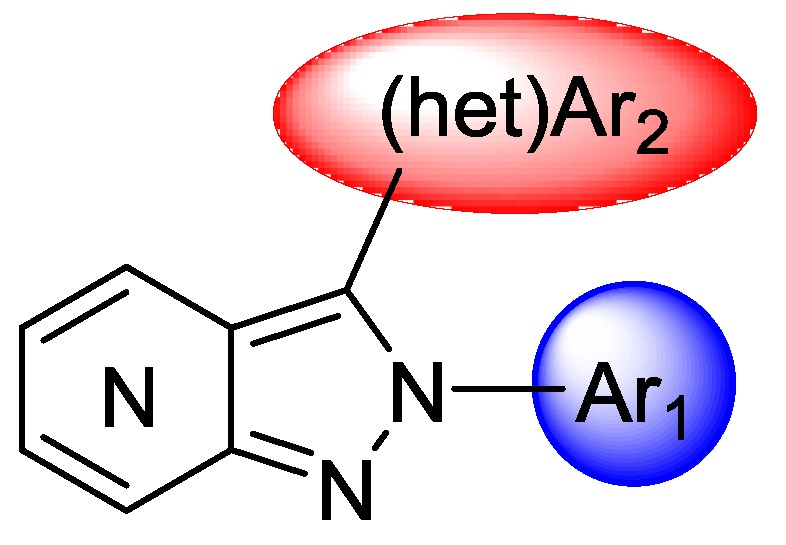


Seventeen compounds were subsequently synthesized, and our best ligand exhibited an IC_50_ of 450 nM against COX-2 (vs. IC_50_ COX-1 > 25 µM). This lead compound has been radiolabelled with ^18^F fluorine. In vivo studies will be realized as soon as possible to validate the potency of the ligand to image COX-2 in animal model.

### 4.2. Scale-up of the Sandmeyer Reaction in a Continuous Safety Process

D’AttomaJoseph^1^BrunPierre-Louis^2^RobinYves^2^BostynStéphane^3^RoutierSylvain^1^BuronFrédéric^1^*1Institut de Chimie Organique et Analytique, UMR CNRS 7311, Université d’Orléans, rue de Chartres—BP 6759, F-45067 Orléans Cedex 2, France2ISOCHEM, 4 rue Marc Sangnier, BP 16729, F-45300 Pithiviers, France3ICARE, 1C avenue de la Recherche Scientifique CS 50060, F-45071 Orléans Cedex 2, France*Correspondence: frederic.buron@univ-orleans.fr

Diazonium compounds are extremely versatile intermediates in organic synthesis. They have found applications in alkylation or heterocycle-forming cycloaddition reactions and provide access to azo compounds or reactive reagents such as ketenes and carbenes (Maas, G., *Angew. Chem. Int. Ed.* 2009, *48*, 8186–8195; Pechmann, H.V. *Ber. Dtsch. Chem. Ges.* 1894, *27*, 1888–1891; Corey, E.J., et al. *J. Am. Chem. Soc.* 1965, *87*, 2518–2519; Candeias, N.R., et al. *J. Org. Chem.* 2008, *73*, 5926–5932; Ye, T., et al. *Chem. Rev.* 1994, *94*, 1091–1160). Diazo species are also employed as leaving groups in important reactions including Sandmeyer, Meerwein, Balz–Schiemann and palladium-catalyzed cross-coupling (Galli, C., *Chem. Rev.* 1988, *88*, 765–792; Browne, D.L., *Angew. Chem. Int. Ed.* 2014, *53*, 1482–1484; Prasad Hari, D., et al. *Angew. Chem. Int. Ed.* 2014, *53*, 725–728; Abele, S., et al. *Org. Process Res. Dev.* 2014, *18*, 993–1001; Roglans, A., et al. *Chem. Rev.* 2006, *106*, 4622–4643; Kikukawa, K., et al. *Chem. Lett.* 1977, *6*, 159–162; Okazaki, T., et al. *Eur. J. Org. Chem*. 2014, 1630–1644). Published warnings on the use of diazonium ions are related to their potentially hazardous behavior, especially on a large scale. This major drawback is an important obstacle to using diazo compounds in industrial processes.

To use these species on a large scale, we were interested in the development of an innovative technology in organic chemistry: continuous flow synthesis. Flow chemistry offers new opportunities for in situ generation and thus use these highly reactive intermediates at a range of scales under safety conditions (versus traditional batch processes).

The aim of this project is to transfer the usual “batch” Sandmeyer reaction to the continuous flow process. Thus, this communication will present the effectiveness preparation, use and scale-up of (het)aryl-diazonium salts to generate various chlor-containing (het)aryl derivatives.


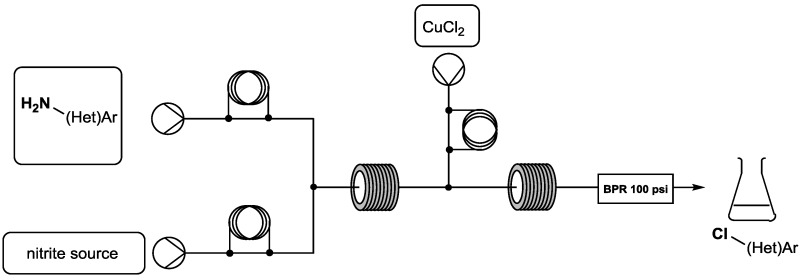


### 4.3. Regioselective Functionalization of New 2, 4-Substituted Pyrido[1′,2′:1,5] pyrazolo[3,4-d]pyrimidines

EjjoummanyAbdelaziz^1^,^2^BuronFrédéric^2^El HakmaouiAhmed^1^RoutierSylvain^2^*GuillaumetGérald^2^*AkssiraMohamed^1^*1Laboratoire de Chimie Physique et Chimie Bioorganique, URA C 22, Pôle RéPAM, F.S.T., Université Hassan II de Casablanca, BP 146 Yasmina, 28800 Mohammedia, Maroc2Institut de Chimie Organique et Analytique, Université d’Orléans, UMR CNRS 7311, F-45067 Orléans Cedex, France*Correspondence: abdelaziz.ejjoummany@univ-orleans.fr

Based on the interest of our group in rare heterocyclic structures (Belaroussi, R., et al. *Synthesis* 2013, *45*, 2557–2566.), we develop novel original synthetic methodologies to design an original heterocycle, pyrido[1′,2′:1,5] pyrazolo[3,4-*d*]pyrimidine. The in situ activation strategy has a long tradition, and the main advantage of this methodology is that it avoids tedious reagent preparation (Kang, F.A., et al. *Eur. J. Org. Chem*. 2009, 461–479). In this context, the use of in situ PyBroP activation (Li, S.M., et al. *Chem. Commun*. 2011, *47*, 12840–12842) followed by cross-coupling reaction allows an easy access to 2,4-disubstituted pyrido[1′,2′:1,5] pyrazolo[3,4-*d*]pyrimidines derivatives. These derivatives were obtained using two orthogonal palladium-catalyzed cross-coupling reactions via successive in situ PyBroP activation and C-2 methylsulfur release. These reactions required a microwave irradiation to reduce the reaction time and enhance yields. Some efforts are in progress to design a bioactive compound from this study.


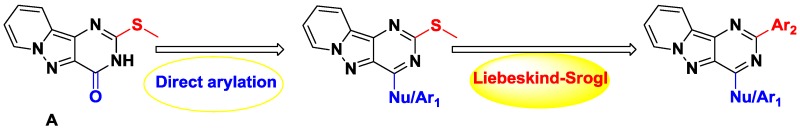


### 4.4. Design, Synthesis, Characterization, Pharmacological Evaluation and In Vivo Study of Harmine Derivatives as New Anticancer Compounds

MarxSébastien*MeinguetCélineWoutersJohanNamur Medicine and Drug Innovation Center (NAMEDIC), Laboratoire de Chimie Biologique Structurale (CBS), Université de Namur (UNamur), 61 rue de Bruxelles, B-5000 Namur, Belgium*Correspondence: sebastien.marx@unamur.be

Harmine **1** is a natural compound that is known for its antiproliferative and anticancer effect. In order to develop new antiproliferative compounds, a three-step strategy has been adopted.

The first part was dedicated to the development and the optimization of three generations of new harmine derivatives **2** trisubstituted on positions 2, 7 and 9, combining a micromolar to submicromolar antiproliferative activity and a high solubility at physiological pH (Frédérick, R., et al., *J. Med. Chem.* 2012, *55*, 6489–6501).

Then, a pharmacological study of the best antiproliferative compound **3** has underlined the selectivity for cancer cells compared to healthy cells and the plasmatic stability in vitro. However, the anticancer property study on a metastatic murine model, after intraperitoneal injection, has highlighted the lack of effect of **3**. One of the given hypotheses is the poor permeability of **3**. In order to administer the compound by intravenous injection and thus enable a better absorption than intraperitoneal injection, two strategies were used (Meinguet, C., et al., *Eur. J. Med. Chem.* 2015, *94*, 48–55).

First, a formulation study with complexation to cyclodextrins **4** has been considered. This study has shown an important increased solubility of **3** in the presence of cyclodextrin while maintaining its submicromolar activity (Meinguet, C., et al., *Eur. J. Pharm. Sci.* 2015, *77*, 135–140). Besides, a derivatization of compound **3** was used. Derivatives **5 A-B-C** including a 2-methylpyridine substituent were synthesized and characterized in order to increase the solubility with pyridine moiety and maintain antiproliferative activity. In particular, compound **5C** has shown an antiproliferative activity similar to compound **3**. Nevertheless, this molecule has shown a kinetic solubility comparable to **3**. As a prospective new formulation, compound **3** will be studied in vivo and the solubility of derivative **5C** could be measured in aqueous solution for intravenous administration (thermodynamic solubility).


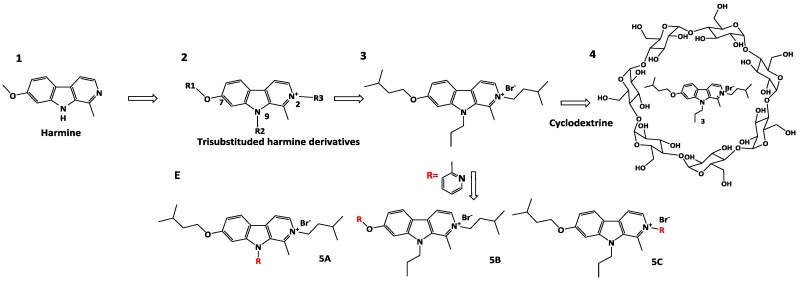


### 4.5. Identification of Chemosensitizing Compounds, Inhibiting the Resistance to Etoposide Induced by TMEM45A Protein in HepG2 Cells Under Hypoxia

MarxSébastien^1^*Dal MasoThomas^1^MichielsCarine^2^WoutersJohan^1^1Namur Research Institute for Life Science (Narilis), Namur Medicine and Drug Innovation Center (Namedic), Laboratoire de Chimie Biologique Structurale (CBS), Université de Namur (UNamur), 61 rue de Bruxelles, B-5000 Namur, Belgium2Namur Research Institute for Life Science (Narilis), Unité de Recherche en Biologie Cellulaire (URBC), Université de Namur (UNamur), 61 rue de Bruxelles, B-5000 Namur, Belgium*Correspondence: sebastien.marx@unamur.be

The development of resistance to chemotherapy continues to be a major problem in the treatment of cancer patients. Tumor microenvironment, and more specially hypoxia (1% O_2_) is one of the key factors known to favor tumor growth as well as resistance to treatment (Rebucci, M., et al., *Biochem. Pharmacol.* 2013, *85*, 1219–1226).

TMEM45A has been identified as a protein whose expression is increased by hypoxia, which displays strong anti-apoptotic properties and which seems to be involved in the resistance of cancer cells to chemotherapeutic drugs. In particular, when TMEM45A expression was silenced, human hepatocarcinoma cells (HepG2) incubated with etoposide under hypoxia became sensitive to cell death induced by this chemotherapeutic molecule (Flamant, L., et al. *BMC Cancer* 2012, *12*, 391). The aim of this project is to identify chemosensitizing compounds that are able to inhibit the resistance to etoposide induced by TMEM45A protein in HepG2 cells. In order to achieve this goal, a functional test has been developed in order to screen a compound library from Namur Medicine and Drug Innovation Center (Vollenweider, I., et al. *J. Immunol. Methods* 1992, *149*, 133–135). First, the toxicity of a subgroup of compounds (100 molecules), which are representative of the chemical library, was determined in HepG2 cells with the MTT cell viability assay. Then, different parameters of a functional test based on propidium iodide staining of DNA have been optimized and validated: incubation time with etoposide, cellular density and fixation method. The aim then is to screen the non-toxic molecules with the optimized propidium iodide viability test in the presence of etoposide under hypoxia in order to determine if the compounds are potentially chemosensitizers. These compounds could then be used in combination with conventional chemotherapy to enhance their efficacy.

### 4.6. Discovery of Arginase 1 Inhibitor by Screening of Chelator Fragments Library (P29)

PrevostJulien R.C.*Van HamNicolas J.P.KozlovaArinaFrédérickRaphaëlMedicinal Chemistry Research Group (CMFA), Louvain Drug Research Institute (LDRI), Université catholique de Louvain, B-1200 Brussels, Belgium*Correspondence: julien.prevost@uclouvain.be

Different novel strategies are developed to fight cancer. Among then, immunotherapy, which consists of the stimulation of the patient’s own immune system against cancer cells is very promising (King, J., et al. *QJM* 2008, *101*, 675–683). This strategy has numerous advantages such as minimal side effects, and the possibility to exert a durable therapeutic effect due to the induction of immunologic memory. However, this strategy is also hampered by some limitations. Among these is the fact that cancer cells are able to develop a number of mechanisms to escape immune rejection (Muller, A.J., et al. *Nat. Rev. Cancer* 2006, *6*, 613–625). The understanding of these resistance mechanisms could facilitate the search for effective vaccines. Different mechanisms allowing tumors to escape the immune system have already been suggested and involve several enzymes such as indoleamine 2,3-dioxygenase IDO), tryptophan 2,3-dioxygenase (TDO), induced oxide nitric synthase (iNOS), and arginase 1 (ARG-1) (Grohmann, U., et al. *Immunol. Rev.* 2010, *236*, 243–264). Arginase 1 is a key metalloenzyme involved in the urea cycle where it converts L-arginine to L-ornithine and urea (Ash, D.E. *J. Nutr.* 2004, *134*, 2760S–2767S). This enzyme is seen as a promising target in different diseases such as allergic asthma, inflammatory bowel disease, ulcerative colitis, cardiovascular disease, or erectile dysfunction (Ivanenkov, Y.A., et al. *Pharm. Pat. Anal.* 2014, *3*, 65–85). In cancer, the expression of arginase I by myeloid-derived suppressor cells (MDSCs) prevents the formation of the CD3 receptor ζ chain and thus impairs TCR functions because of the depletion of L-arginine concentration in the extracellular environment (Rodriguez, P.C., et al. *Cancer Res.* 2004, 5839–5849; Gabrilovich, D.I., et al. *Nat. Rev. Immunol.* 2009, *9*, 162–174). This leads to inhibition of T lymphocyte proliferation and avoids the immune response. Inhibitor of ARG-1 would thus be very interesting to restore the homeostasis of l-Arginine required for the immune response (Di Costanzo, L., et al. *Proc. Natl. Acad. Sci. USA* 2005, *102*, 13058–13063).

New strategies on metalloproteins focus on targeting their metallic cations. Building up the Chelator Fragments Library (CFL) would allow identification of new and interesting scaffolds binding to the proteins (Agrawal, A., et al. *ChemMedChem* 2010, *5*, 195–199; Jacobsen, J.A., et al. *J. Med. Chem.* 2011, *54*, 591–602) and discovery of new classes of inhibitors (Rouffet, M., et al. *Dalt. Trans.* 2011, *40*, 3445–3454; Rouffet, M., et al. *J. Am. Chem. Soc.* 2010, *132*, 8232–8233). A structure-based drug design in relation to this new approach focusing on metal interactions was developed in our laboratory. In this poster, we will present our current strategy as well as our advancements in the development of ARG1 inhibitors (Shin, H., et al. *J. Am. Chem. Soc.* 2004, *126*, 10278–10284; Cama, E., et al. *J. Am. Chem. Soc.* 2003, *125*, 13052–13057).

### 4.7. Design, Synthesis and Pharmacological Evaluation of Dimeric Ligands for the Benzothiadiazine Dioxide Allosteric Binding Site of the α-Amino-3-hydroxy-5-methyl-4-isoxazolepropionic Acid (AMPA) Receptors

DrapierThomas^1^*GeubellePierre^1^,^2^BouckaertCharlotte^3^GoffinEric^1^DillySébastien^1^HansonJulien^1^,^2^PochetLionel^3^KastrupJette Sandholm^4^FrancottePierre^1^PirotteBernard^1^1CIRM-Medicinal Chemistry, Université de Liège, Avenue Hippocrate, 15, B36, B-4000 Liège, Belgium2GIGA-Molecular Pharmacology, Université de Liège, Avenue Hippocrate, 11, B-4000 Liège, Belgium3NAmur MEdicine and Drug Innovation Center (NAMEDIC), FUNDP, rue de Bruxelles 61, B-5000 Namur, Belgium4Department of Drug Design and Pharmacology, University of Copenhagen, Universitetsparken 2, DK-2100 Copenhagen, Denmark*Correspondence: Thomas.drapier@ulg.ac.be

l-glutamic acid is the major excitatory neurotransmitter in the brain. It exerts its effects through metabotropic and ionotropic receptors. Among the latter, three subtypes have been identified: *N*-methyl-d-aspartate (NMDA), α-amino-3-hydroxy-5-methyl-4-isoxazolepropionic acid AMPA) and kainite (KA) receptors. It is now well established that a deficit in glutamatergic signaling may be responsible for neurological disorders such as mild cognitive impairment, schizophrenia, depression, and ADHD. Enhancement of the signal through positive allosteric modulators of AMPA receptors might be a therapeutic issue for these diseases. These compounds are expected to exert a fine tuning of the signal. Since they require the presence of the endogenous ligand to be active, they are expected to induce less toxicity than agonists.

In this context, based on the structure of known allosteric modulators of AMPA receptors such as cyclothiazide and IDRA 21, the Laboratory of Medicinal Chemistry (University of Liège) has developed a series of 1,2,4-benzothiadiazine 1,1-dioxides with high potency as AMPA receptor potentiators (Krintel, C., et al. *Biochem. J*. 2012, *441*, 173–178; Nørholm, A.B., et al. *J. Med. Chem*. 2013, *56*, 8736–8745). Crystallographic data obtained by the Department of Drug Design and Pharmacology (University of Copenhagen) highlighted how those potentiators bind to two contiguous sites at the dimer interface of the ligand-binding domain (LBD) of the AMPA receptor 1,2. Based on these data, we may expect that the synthesis of dimeric molecules could lead to further improvement in affinity and activity. This assumption was reinforced by docking experiments conducted with virtual examples of dimeric compounds on the GluA2-LBD (collaboration with NAMEDIC).

The present work thus focuses on the preparation of a family of dimeric benzothiadiazine dioxides, responding to general structure (1). Moreover, in collaboration with GIGA-Molecular Pharmacology, we are developing a pharmacological in vitro assay based on the measurement of Ca^2+^-inflow through a fluorimetric method. This medium-throughput screening will enable the characterization of our new compounds and the validation of our working hypothesis.


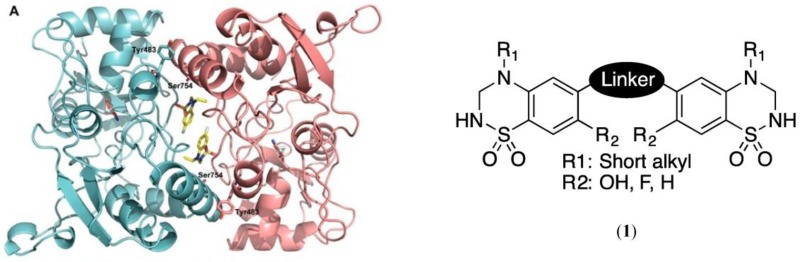


### 4.8. Structural Study of Membrane Protein TMEM45A Involved in the Resistance of Cancer Cells against Chemotherapeutic Agent

Dal MasoThomas^1^*MarxSébastien^1^MichielsCarine^2^WoutersJohan^1^1Laboratoire de Chimie Biologique Structurale, Université de Namur (UNamur), B-5000 Namur, Belgium2Unité de recherche en biologie cellulaire animale (URBC), Université de Namur (UNamur), 5000 Namur, Belgium*Correspondence: thomas.dalmaso@unamur.be

Cancer is the leading cause of death worldwide. The development of therapy resistance continues to be a major problem in the treatment of patients with cancer. Multiple processes influence tumor response to therapies. Treatment failure has been identified as one of the four major issues in cancer research. Recently, we developed new cellular models to demonstrate that tumor microenvironment and especially hypoxia promotes tumor growth and resistance to treatment. In this light, we have identified the protein TMEM45A. Its expression is increased under hypoxic conditions and its overexpression shows anti-apoptotic properties as well as resistance of cancer cells to chemotherapeutic agents. Indeed, the silencing of TMEM45A in hepatocellular carcinoma cells (HepG2) incubated in the presence of etoposide under hypoxic conditions results in a sensitization of the latter to cell death induced by the anticancer agent (Flamant, L., et al. *BMC Cancer* 2012, *12*, 391–407; Hayez, A., et al. *Exp. Dermatol.* 2014, *23*, 339–344; Cosse, J.P., et al. *Anti-Cancer Agents Med. Chem.* 2008, *8*, 790–797).

This work focuses on the structural study (protein topology and 3D structure) of membrane protein TMEM45A. The aim is to improve the understanding of structure and function of the protein allowing identification of chemosensitizer agents capable of enhancing the action of chemotherapeutic compounds.

In order to study the membrane topology, we will use molecular biology techniques (insertion and fusion mutants) but also the compartmentalization property of glycosylation. Overexpression, purification and crystallization (in lipidic cubic phase) of the protein will also be carried out to obtain the 3D structure. Structural informations will allow virtual screening of a chemical library and identification of a potential ligand as well as new insights on the TMEM45A function and its involvement in the resistance mechanism to apoptosis induced by etoposide.

### 4.9. Design, Synthesis, and Biological Activity of Pyridopyrimidine Analogs as Potent Kinase Inhibitors for Neurodegenerative Diseases

ChampiréAnthony*PléKarenRoutierSylvainInstitut de Chimie Organique et Analytique, UMR CNRS 7311, Université d’Orléans, F-45067 Orléans Cedex, France*Correspondence: anthony.champire@etu.univ-orleans.fr

Pyridopyrimidines are a very well-known class of nitrogen heterocycles with a high potential in medicinal chemistry. Indeed, they have been studied for their properties against several diseases such as cancer, hepatitis or neurodegenerative disorders (Daub, H., et al. PCT Int. Appl. WO 2005105097 A2, 2005). Due to their diverse activities, we decided to use this scaffold to develop new molecules for the treatment of neurofibromatosis, an orphan disease which affects 1 in 3500 people worldwide.

Our major interest is the synthesis of new pyrido[3,2-*d*]pyrimidine derivatives as potential kinase inhibitors. We have previously described the synthesis of a new library of 2,7 disubstituted pyrido[3,2-*d*]pyrimidines targeting DYRK1A and CDK5 giving derivative **I** as one of the most active compounds (Dehbi, O., et al. *Eur*. *J*. *Med*. *Chem*. 2014, *80*, 352–363). A 2,4 disubstituted pyrido[3,2-*d*]pyrimidine **II** inhibiting PI3K and mTOR has also been described (Saurat, T., et al. *J. Med. Chem.* 2014, *57*, 613−631). These two families have proven their efficiency and encouraged us to design a new series of other 2,4 disubstituted pyrido[3,2-*d*]pyrimidine **III** to target the LIMKs, kinases deregulated in patients affected by neurofibromatosis. In parallel we have developed a synthetic strategy to selectively obtain two different isomers of tricyclic triazolopyridopyrimidine compounds **IV** and **V** via an original use of the Dimroth rearrangement.

To date, a new library of original pyridopyrimidines **III** has been synthetized and is currently being tested on a kinase panel including LIMK2. A first library of tricyclic compounds **IV** and **V** have also been synthesized as building blocks for the preparation of bioactive molecules. Development of a new series of triazolopyridopyrimidine analogs targeting the LIMKs is in progress.


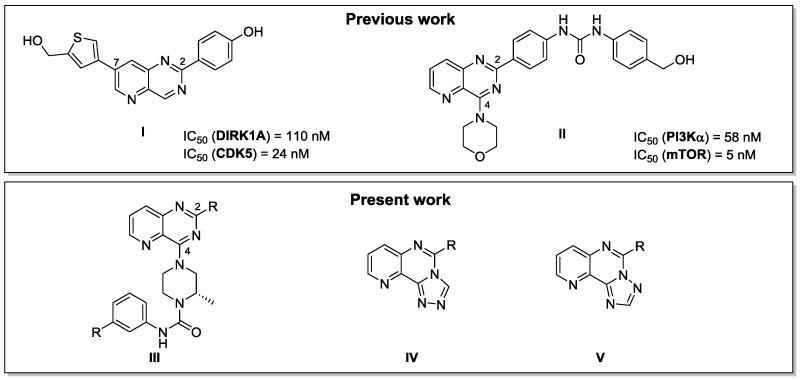


## 5. Conclusions

At the end of the meeting, the following awards were presented to honor the work of the PhD Students or young scientists:

- Prize for the best oral communication: Romain Duroux (University of Lille) entitled “From Design to Pharmacological Evaluation of Benzoxazole Derivatives as Adenosine A_2A_ Receptor Antagonists for the Potential Treatment of Alzheimer Disease” and Quentin Spillier (University of Louvain) for his presentation “undisclosed summary.”
- Prize for the best poster presentation: Jonathan Elie (University of Tours) “Conception and Evaluation of Fluorinated Heterocyclic Compounds as COX-2 Ligands to Visualize and Quantify Neuroinflammation by PET.”
- Prize of the Journées Franco-Belges de Pharmacochimie: This prize is awarded to a young scientist (PhD student) with outstanding curriculum vitae, to encourage them to continue their scientific career. This year the prize goes to G. Berger of the University of Brussels.

In 2017, the “31ièmes Journées Franco-Belges de Pharmacochimie” are to take place in Belgium.

